# Identification of visible and near-infrared signature peaks for arboviruses and *Plasmodium falciparum*

**DOI:** 10.1371/journal.pone.0321362

**Published:** 2025-04-17

**Authors:** Brendon Goh, Ricardo J. Soares Magalhães, Silvia Ciocchetta, Wenjun Liu, Maggy T. Sikulu-Lord

**Affiliations:** 1 School of the Environment, Faculty of Science, The University of Queensland, Brisbane, Australia; 2 UQ Spatial Epidemiology Laboratory, School of Veterinary Science, The University of Queensland, Brisbane, Australia; 3 Australian Defence Force, Malaria and Infectious Disease Institute, Brisbane, Australia; Institute of Tropical Medicine: Instituut voor Tropische Geneeskunde, BELGIUM

## Abstract

Arbovirus and malaria infections affect more than half of the world’s population causing major financial and physical burden. Current diagnostic tools such as microscopy, molecular and serological techniques are technically demanding, costly, or time consuming. Near-infrared spectroscopy has recently been demonstrated as a potential diagnostic tool for malaria and Dengue virus and as a screening tool for disease vectors. However, pathogen specific absorption peaks that allow detection of these infections are yet to be described. In this study, we identified unique visible and near-infrared peaks from existing laboratory strains of four major arboviruses including Barmah Forest virus, Dengue virus, Ross River virus, Sindbis virus and *Plasmodium falciparum*. Secondly, to determine the diagnostic ability of these peaks, we developed machine learning algorithms using artificial neural networks to differentiate arboviruses from media in which they were grown. Signature peaks for BFV were identified within the visible region at 410, 430, 562 and 588 nm and the near-infrared region at, 946, 958, 1130, 1154 and 1780 nm. DENV related peaks were seen at 410nm within the visible region and 1130 nm within the near-infrared region. Signature peaks for Ross River virus were observed within the visible region at 410 and 430 nm and within the near-infrared region at 1130 and 1780 nm, while Sindbis virus had a prominent peak at 410 nm within the visible region. Peaks at 514, 528, 547, 561, 582, and 595 nm and peaks at 1388, 1432, 1681, 1700, 1721, 1882, 1905, 2245, 2278, 2300 nm were unique for *P. falciparum*. Near-infrared spectroscopy predictive sensitivity defined as the ability to predict an arbovirus as an infection was 90% (n=20) for Barmah Forest virus, 100% (n=10) for Ross River virus and 97.5% (n=40) for Dengue virus, while infection specificity defined as the ability to predict media as not-infected was 100% (n=10). Our findings indicate that spectral signatures obtained by near-infrared spectroscopy are potential biomarkers for diagnosis of arboviruses and malaria.

## Introduction

Arboviruses persist in nature through a life cycle involving a vertebrate host, an organism that carries the virus and an infected arthropod, usually mosquitos or ticks [1]. Vector borne diseases have been on the rise due to increased geographical distribution and abundance of arthropod vectors mainly because of concurring factors such as climate change, migration and urbanization [2]. For instance, infection due to Dengue virus (DENV) is now considered the most common vector-borne infection globally as it affects more than half of the world’s population [3]. In 2023, over 6.5 million cases of DENV were reported, resulting in around 6,800 fatalities. The highest incidence was observed in areas of South America and Southeast Asia [4]. Malaria is a mosquito-borne disease caused by the *Plasmodium* parasite which is transmitted to humans through bites of infected female *Anopheles* mosquitoes. In 2023, an estimated 263 million malaria cases and 597,000 malaria related deaths were reported by the World Health Organisation highlighting malaria as a major public health concern [5]. Traditionally, malaria is diagnosed using microscopy and Giemsa-stained blood smears [6]. However, with a limit of detection of >50 parasites/μL of blood, it requires a well-trained microscopist [7]. Rapid diagnostic tests are also common for malaria diagnosis because they are very easy to use and do not require qualified personnel, but their sensitivity and specificity is low in detecting low parasitaemia [8,9].

Current diagnosis of arboviruses include molecular methods such as reverse transcriptase-polymerase chain reaction (RT-PCR) [10] and serological techniques such as Enzyme-linked immunosorbent assay (ELISA) [11,12]. Molecular methods are the most accurate and sensitive. For example, RT-PCR for DENV has detection limits that vary between 10–100 copies/reaction depending on the DENV serotype being tested. ELISA assay for detection of DENV-2 specific antibodies has been reported to be 90% (n=20) specific and 100% sensitive (n=20) in 40 human serum samples [13]. Despite high sensitivity, these methods are restricted to the laboratory settings, can be time consuming and costly for programmatic diagnosis and surveillance purposes. Molecular diagnosis techniques for malaria such as Polymerase Chain Reaction (PCR), quantitative-PCR, nested PCR and ELISA have been developed [14–16] but they are time consuming, costly and require trained personnel. Rapid diagnosis tests for malaria are based off the detection for HRP2 antigens as recommend by the World Health organisation [17]. In a recent study, rapid diagnosis test (CareStart™ Malaria HRP2 AccessBio kit) and microscopy both failed to detect more than 40% of infections identified by varATS qPCR [18]; indicating the necessity of novel tools to ensure accurate and prompt diagnosis of clinical malaria cases.

Near-infrared spectroscopy (NIRS) is a potential novel diagnostic/surveillance tool for arboviruses. It involves the interaction of near-infrared light with biological samples to produce a reflectance spectrum [19]. Based on chemical and structural differences between biological samples, unique spectra are produced. The spectra reflect the amount and type of biochemical composition of the sample and can be used to typify those samples. NIR spectra can provide insights into functional groups of samples, such as C–H, O–H, and N–H. Any molecule that contains hydrogen will exhibit a detectable NIR spectrum, making a wide variety of biological substances appropriate for NIR analysis [20]. NIRS can therefore be used as a biomarker for biological samples. Moreover, NIRS is rapid, non-invasive, and does not require skilled personnel to operate, enhancing its effectiveness in the field and in areas lacking scientific equipment.

To date, four studies have shown the ability of NIR technique to detect DENV, Chikungunya, *Wolbachia* and Zika in *Ae. aegypti* mosquitoes with accuracies above 90% [21,22]. However, only one study has demonstrated absorbance frequencies in the visible region for arboviruses. Firdous and colleagues reported that NIR peaks at 533 and 580 nm are indicative of the presence of the DNA mixture of DENV2 and DENV3 in human blood samples [23]. The application of NIRS for the detection of the *plasmodium* parasite has been reported in several studies [24–26]. These studies identified prominent peaks at 650 nm (mice whole blood) [24], 930–1660 nm (human skin) [25] and 1503–2306 nm (human whole blood) [26]. In this study, we identified NIR biomarkers for Barmah Forest virus (BFV), DENV, Ross River virus (RRV), Sindbis virus (SINV) and purified *P. falciparum*. These signature peaks provide a valuable foundation for diagnosing arboviruses and malaria using NIRS, serving as a basis for future analysis of human or vector samples.

## Materials and methods

### Dengue virus culture

DENV prototype strains DENV-1 Hawaii (1944), DENV-2 NGC (1944), DENV-3 H-87 (1956) and DENV-4 H241 (1956) were used in this study. DENV was propagated in C6/36 *Aedes albopictus* cells, maintained at 28°C in RPMI and supplemented with 10% FBS and 1% PSG. Following three passages in C6/36 cells, virus stocks were concentrated using Ultracel-100k filters (Amicon, Tullagreen, Cork Ireland) [27] and frozen at -80°C until further use.

### Barmah Forest virus and Ross River virus culture

BFV QML and BFV WEN 1631 and RRV QML1 strain (GenBank No. GQ433354) were used in this study. The virus strains were passaged three times in Vero cells, maintained at 37°C in RPMI and supplemented with 10% FBS and 1% PSG. Following three passages in Vero cells, virus stocks were concentrated using Ultracel-100k filters (Amicon, Tullagreen, Cork Ireland) [27] and frozen at -80°C until further use. One vial of the viral stocks was thawed to determine virus titre using 50% tissue culture infectious dose (CCID50/ml) on Vero cells as described [28]. Briefly, virus stocks were 10-fold serially diluted and 100 µL of diluted virus was inoculated onto monolayers of Vero cells grown in 96 well plates in cell culture media and maintained at 37°C, 5% CO_2_. Ninety-six hours later, cells were fixed with 3.7% formaldehyde, stained with 1% crystal violet for 1 hour, washed in tap water and dried. The cell culture infectious dose 50% was determined from titration endpoints as described elsewhere [29] and expressed as the Vero cell CCID50/mL.

### Sindbis virus culture

SINV strain (SINV 18953) was propagated in C6/36 *Ae. albopictus* cells, maintained at 28°C in RPMI and supplemented with 10% FBS and 1% PSG. Following three passages in C6/36 cells, virus stocks were concentrated using Ultracel-100k filters (Amicon, Tullagreen, Cork Ireland) and frozen at -80°C until further use. One vial of the viral stocks was thawed to determine virus titre by 50% tissue culture infectious dose (CCID50/ml) on Vero cells as described by Sudeep et al [30]. Briefly, virus stocks were serially diluted 10-fold and 100 µL of diluted virus was inoculated onto monolayers of Vero cells grown in 96 well plates in RPMI 1640 supplemented with L-glutamine, 5% FBS, 1% PSG and maintained at 30°C, 5% CO_2_. After 4 days of incubation, cells were fixed with 3.7% formaldehyde, stained with 1% crystal violet for 1 hour, washed in tap water, dried, and counted. The CCID50/ml were calculated according to published Reed-Muench method [31].

### Virus free media controls

Virus free cell culture media used in this study consisted of sterilised Roswell Park Memorial Institute Medium (RPMI), 1640 Medium (Sigma Life Sciences, USA) with 10% heat-inactivated fetal bovine serum (FBS) (Thermo Fisher Scientific, USA) and 1% Penicillin-Streptomycin Glutamine solution (PSG) (Thermo Fisher Scientific, USA).

### Arboviruses viral titer determination for NIRS spectral collection

All arboviruses used were passaged in 3 separate batches. Samples from each batch were used as a single biological replicate (Table 1). Virus stocks were titrated using a modification of the Enzyme-linked immunosorbent assay procedure of Broom et al. [32]. Briefly, virus stocks and samples were serially diluted 10-fold and inoculated onto monolayers of C6/36 cells grown in C6/36 cell culture media which consisted of RPMI, 1640 Medium (Sigma Life Sciences, USA) with 5% heat-inactivated FBS (Thermo Fisher Scientific, USA) and 1% PSG (Thermo Fisher Scientific) and maintained at 30°C, 5% CO_2_. After 7 days of incubation, cells were fixed in acetone: methanol (1:1) for 1 hour at 4°C. Plates were air-dried and antigen was detected using a cocktail of anti-flavivirus monoclonal antibody hybridoma supernatants; 4G2 [33] 6B-6C1:3H5 [34], at a ratio of 1:1:1, followed by horseradish peroxidase (HRP-) conjugated goat anti-mouse polyclonal antibody (DAKO, Carpinteria, CA, USA) (1:2000 in PBS-Tween 20). Antibodies bound to the cell mono-layers were detected by the addition of 3,3’,5,5’-tetramethylbenzidine liquid substrate system for membranes (Sigma-Aldrich). The CCID50 was determined from titration endpoints as described elsewhere [29] and expressed as C6/36 CCID50/mL. This experiment was repeated three times at three separate time points to create three independent biological replicates (Table 1).

**Table 1:** A summary of the titres of arboviruses in 3 separate titration batches. Each batch represents a biological replicate grown and analysed at a different time point. Titre is expressed as Median Tissue Culture Infectious Dose (TCID50)/ml with the units Virus Particle (VP)/mL.

Arbovirus	Titre (VP/mL)		
	Biological replicate 1	Biological replicate 2	Biological replicate 3
BFV QML	10^7.75^	10^7.75^	10^9.5^
BFV WEN	10^8.3^	10^7.25^	10^8.3^
RRV QML1	10^7.8^	10^8.25^	10^8.25^
SINV 18953	10^8.5^	10^8.5^	10^8.5^
DENV1 EM-093	10^6.75^	10^6.5^	10^6.5^
DENV2 NGC	10^7.6^	10^7.6^	10^7.6^
DENV3 44002	10^6.3^	10^6.3^	10^6.3^
DENV4 AFRIM	10^6.6^	10^6.6^	10^6.6^

### Ethics

Five biological replicates of serum samples each consisting of 150 mL of pooled human serum samples were obtained from Australian Red Cross Lifeblood using human ethics protocol approved by The University of Queensland (Ethics approval number 2020001077, from 10 July 2020 to 31 August 2023). Participants were recruited beginning on 8 June 2023 to 22 June 2023. Following collection from donors, all samples were routinely tested for Hepatitis B and C, HTLV I/II, Syphilis HIV 1/2, and ABO/Rh antigens. Human serum was stored at -25°C for 3–4 days prior to running the described experiments to preserve proteomic profile integrity and was thawed fully at room temperature before use. All samples supplied by the Australian Red Cross Lifeblood were fully anonymized before handover.

### *P. falciparum* cell culture and *Plasmodium* free media

Base media prepared for *P. falciparum Welch* was ATCC medium 2196 - Malaria medium (American Type Culture Collection, USA) which consist of a sterilized mixture of RPMI-1640 (Sigma R-0883), HEPES buffer (1 M), Gentamicin (50 mg/ml), L-glutamine (100 mM), Hypoxanthine (100 mM), Glucose (20%) and NaOH (1 N). The mixture was sterilised by filtering through 0.22 μM Millex® filter (Millipore, USA). Complete medium was made by adding the heat-inactivated (at 56°C for 1 hour) human serum to 10% (vol/vol) to the base medium and was used to culture parasites as previously described [35]. *P. falciparum Welch* strain FCR-3/FMG (American Type Culture Collection, USA) had an initial concentration of 255 parasites/mL. The base media alone was used as the control.

### Spectra collection and analysis

LabSpec 4 near-infrared spectrometer (ASD Malvern Panalytical, Malvern, United Kingdom) was used to scan all samples. Details of the spectrometer used is published elsewhere [36]. RS^3^ software (Malvern ASD Panalytical) was used for NIRS spectra collection. Baseline calibration and optimization were done at the beginning of each experiment and every 30 minutes by scanning an empty space on the glass slide placed on a white Spectralon plate. Five µL of each arbovirus, *P. falciparum*, and respective media were aliquoted onto glass microscope slides to obtain a sample. A total of 10 technical replicates were scanned for each biological replicate of arbovirus, *P. falciparium* and media. Samples were scanned at approximately 2 mm from the light source by pointing the probe down to the centre of the sample for approximately 3–5 seconds. Full visible and NIR spectra was collected in the 350–2500 nm range of which 350–750 nm and 751–2500 nm belong to the visible light and NIR light regions, respectively. 

### Artificial neural network predictive analysis

Reflectance spectra were converted to absorbance using the formular Log $\frac{1}{R}$. All spectral signatures were converted from txt to csv in ViewSpecPro software (Analytical Spectral Devices Inc, Boulder, CO, USA). To identify arbovirus and *Plasmodium* peaks of importance, raw spectra was converted into 2^nd^ derivative using the Savitzky–Golay [37] with 2^nd^ order smoothing by combining 10 neighbouring data points. 2^nd^ derivative spectra graphs were visualised in GraphPad Prism 9 (GraphPad Software, Inc, California, USA). In the 2^nd^ derivative graph, the difference between pathogen positive and negative samples is shown and unique absorption peaks for pathogens are identified. Model screening and data analysis were conducted in JMP Pro 16 software on raw data (SAS Institute Inc., Cary, NC, USA). Spectra of DENV1, DENV2, DENV3 and DENV4 were combined into a single identifier referred to as DENV. Likewise, spectra of BFV QML strain and BFV WEN 1631 strain were combined into a single identifier referred to as BFV. This is because no differences were observed between strains of these arboviruses (Table S1). SINV data was analysed separately due to spectral outliers that misclassified other arboviruses. Unsupervised learning using principal component analysis and discriminant analysis (PCA-DA) were conducted on all raw spectra as an initial differentiation step for visualisation.

**Fig 1:**
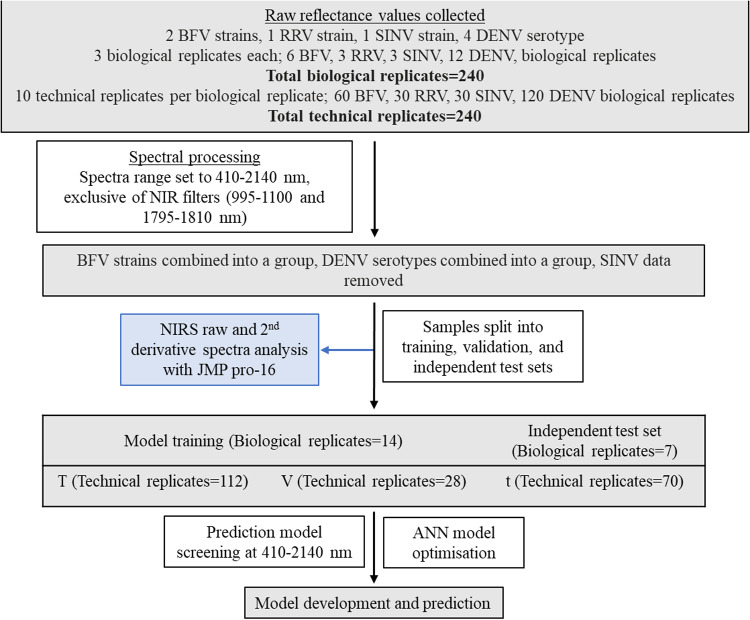
Flow diagram of steps taken to conduct data analysis. The flow of information from data collection to analysis including the number of samples used for training, validation and test sets. Training and validation sets were split based on biological replicates. ‘T’ represents training set, ‘V’ represents validation set and t represents the test set.

The spectral data was first split into two groups: model training/validation (consisting of 16 biological replicates) and an independent test set (consisting of 8 biological replicates). The training (T), validation (V) and test sets (t) were separate biological replicates grown and analysed at different time points. Appropriate machine learning algorithm for each raw spectral data underwent model screening where the following model types were screened for preliminary accuracy: Bootstrap Forest [38], Naïve Bayes [39], Artificial Neural Network (ANN) [40] and Support vector machines [41] (Table S2). ANN produced the most accurate preliminary results and was therefore selected for further analysis. Spectral signatures from 410 to 2140 nm were used. This region was exclusive of spectral noise between 350–409 and 2141–2500 nm and NIR transition filters at 995–1100 and 1795–1810 nm. Spectra were used as model predictors whereas infection status (positive or negative) was used as the response factor. ANN model was developed using random K-Fold cross-validation (n=5 samples). The Neural Networks consisted of one layer with three TanH activation nodes boosted at a learning rate of 0.1 iteratively for 100 tours. Models were built to differentiate arboviruses from media. A summary of sample distribution between training, validation and independent test set is shown in Fig 1.

## Results

### 2^nd^ derivative spectra for arboviruses

**Fig 2:**
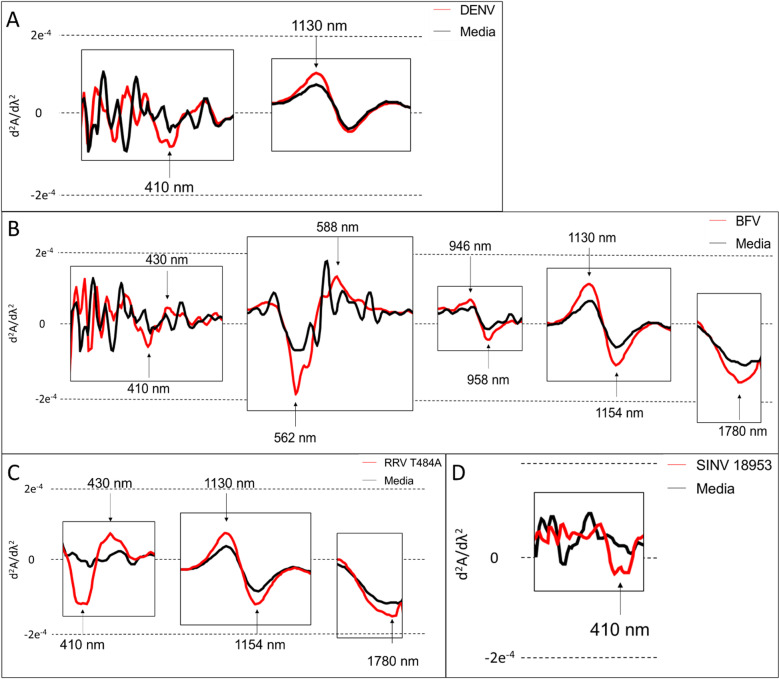
The average 2^nd^ derivative of visible and NIR spectra highlighting prominent peaks for DENV (A), BFV (B), RRV (C) and SINV (D) relative to media. Peaks of importance are shown with black arrows.

Signature peaks for DENV are seen at 410 nm within the visible region and at 1130 nm within the NIR region. A DENV peak is observed to have lower absorbance than media at 410 nm but a higher absorbance value than media at 1130 nm (Fig 2A).

A total of 10 prominent peaks were identified for BFV. These prominent peaks are within the visible region at 410, 430, 562 and 588 nm and within the NIR region at 946, 958, 1130, 1154, 1287–1331 and 1780 nm. Absorbance peaks within the visible region at 430 and 588 nm and within the NIR region at 946, 1130 nm have higher absorbance values than media while peaks at 410 and 562 nm in the visible region and 958, 1154 and 1780 nm in the NIR region have lower absorbance values than media (Fig 2B).

Prominent peaks for RRV were identified at 410 and 430 within the visible region and at 1130, 1154, 1447, 1464 and 1780 nm within the NIR region. Overall, RRV was observed to have 7 prominent peaks (Fig 2C).

The 2^nd^ derivative NIR spectra of SINV 18953 showed prominent peaks at 410, 1447 and 1463 nm. SINV had the lowest number of specific prominent peaks (3 peaks) compared to the other arboviruses (Fig 2D).

### Summary of DENV, BFV, SINV and RRV signature NIR peaks

Four pathogen related wavelengths fell within the visible light region. Two of which (410 and 430 nm) are within the blue visible region and the other two (562, 588 nm) are seen within the green visible light spectrum. BFV prominent peaks at 946 and 958 nm were observed in the 3^rd^ overtone region. Three pathogen related prominent peaks (1130, 1154 and 1780 nm) were seen in the 2^nd^ overtone region. Peaks at 410, 430, 1130 and 1780 nm were observed in BFV, DENV, RRV and SINV. Only one signature peak was identified for SINV which fell within the visible light region, the single signature peak was also identified in all the other arboviruses. Based off this result, we excluded SINV in the analysis. A summary of unique peaks identified for all arboviruses relative to published literature is shown in Table 2.

**Table 2:** Summary of unique 2^nd^ derivative arbovirus peaks, their respective functional group or vibration and representation from literature [42–48].

Wavelength (nm)	Arbovirus	Wavelength region	Functional group/ vibration
410	BFV strains, DENV strains, RRV and SINV	Visible light	Blue visible light
430	BFV strains and RRV		
562	BFV strains		Green visible light
	588		
946	BFV strains	3^rd^ overtone region	CH_2_
958			
1130	BFV strains, DENV strains and RRV	2^nd^ overtone region	CH_3_
1154	BFV strains		
1780	BFV strains and RRV		C=O

### Differentiation of BFV, DENV, RRV, SINV and Vero media using unsupervised machine learning

**Fig 3:**
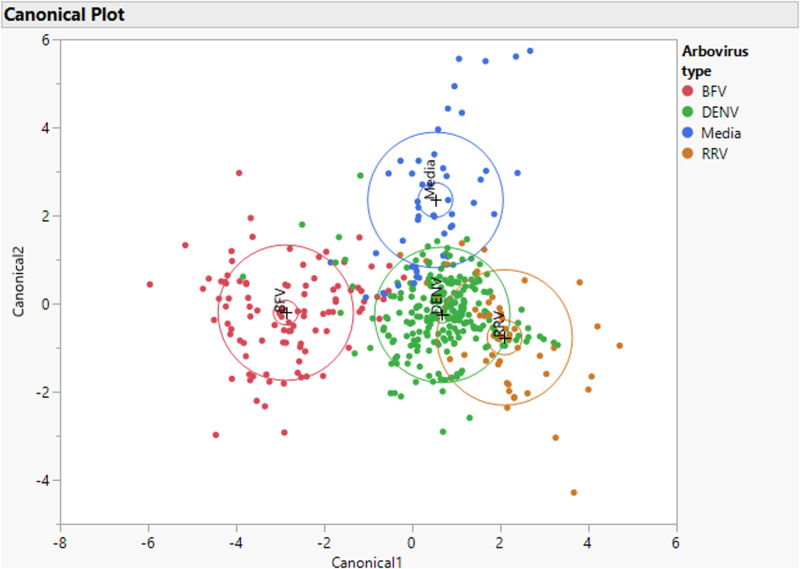
A Canonical plot showing the separation of BFV (Red), DENV (Blue), RRV (Green) and Vero media (Brown). Crosses in the plot represent the average while larger circles represent the standard deviation of data points.

For initial differentiation of BFV, DENV, RRV and Vero media, we attempted to use PCA-DA using the 350–2500 nm region. PCA-DA differentiated arboviruses and media with a misclassification rate of 15.1% and R square value of 0.676 (n= 430) (Fig 3). The canonical plot grouped DENV and RRV more closely with almost half the data points overlapping. DENV had slight overlaps with Vero media. BFV was distinct from all other tested categories.

### Differentiation of BFV, DENV, RRV, SINV and Vero media using supervised machine learning

To identify if supervised machine learning could differentiate arboviruses from Vero media samples using raw spectra, a model using ANN was applied on the raw spectra between 410–2140 nm (Table 3). Overall, the ANN model differentiated BFV, DENV, RRV and Vero media from each other with an R square value of 1 for both training (n=128) and validation (n=32) set (Table 3). The independent test set consisting of 80 NIR spectra was predicted using the training model. Overall, positive predictive rate defined as the proportion of samples predicted as positive out of those that were truly positive was 100% indicating all infected samples were predicted as infected. The negative predictive rate defined as the proportion of samples predicted as negative out of all those that were truly negative was 76.9% (n=13) meaning some negative samples were predicted as postive. Specificity defined as the proportion of samples predicted as negative out of all negative samples was 100% (n=10) meaning all media samples were predicted as not infected. A summary of technical replicates for the training, validation and test set is shown in Table 3. And a summary of positive prediction rate, negative prediction rate, sensitivity, and specificity of BFV, RRV and DENV for the test set is shown in Table 4.

**Table 3:** A confusion matrix showing the accuracy of differentiating arboviruses from each other and from media for the training, validation, and independent test sets.

Actual	BFV	RRV	DENV	Media
Training set	Prediction count			
BFV (n=32)	32	0	0	0
RRV (n=16)	0	16	0	0
DENV (n=64)	0	0	64	0
Media (n=16)	0	0	0	16
Validation set	Prediction count			
BFV (n=8)	8	0	0	0
RRV (n=4)	0	4	0	0
DENV (n=16)	0	0	16	0
Media (n=4)	0	0	0	4
Test set	Prediction count			
BFV (n=20)	14	1	3	2
RRV (n=10)	0	10	0	0
DENV (n=40)	7	0	32	1
Media (n=10)	0	0	0	10

**Table 4:** A summary of positive prediction rate, negative prediction rate, sensitivity, and specificity of BFV, RRV and DENV. “n” represents the number of technical replicates.

Arbovirus Type	Positive predictive rate	Negative predictive rate	Actual* sensitivity	Overall** sensitivity	Infection specificity
BFV	100% (n=67)	76.9% (n=13)	70% (n=20)	90% (n = 20)	100% (n=10)
RRV			100% (n=10)	100% (n =10)	
DENV			80% (n=40)	97.5% (n= 40)	

*Indicates the proportion of infected samples in the indicated group that were predicted into the correct infection group. For example, the proportion of BFV that were predicted as BFV.

**Indicates the proportion of infected samples in the indicated group that were predicted as infected. For example, the proportion of BFV that were predicted as infected.

### Raw spectra for *P. falciparum*

**Fig 4:**
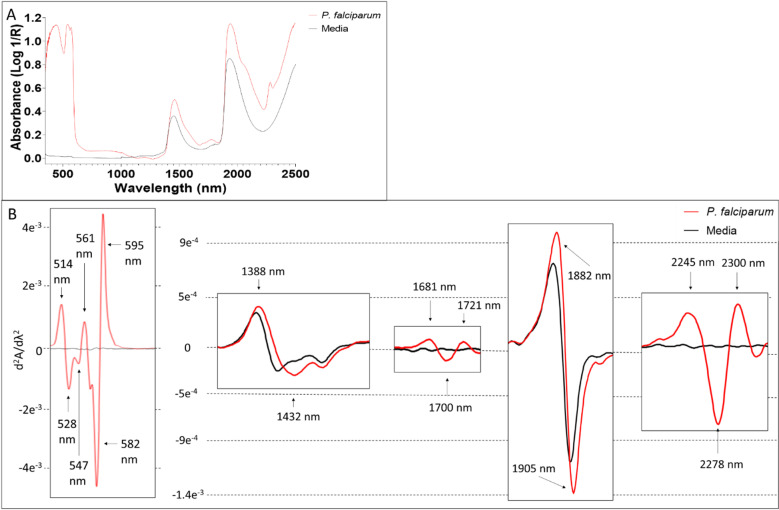
The average raw visible/NIR spectra (A) and the 2^nd^ derivative spectra (B) of *P. falciparum* and media. Peaks of importance are indicated with black arrow.

Unique NIR peaks of *P. falciparum* were observed in the 350–650nm visible region and 1450, 1960 nm NIR region (Fig 4A). Relative to media, a unique peak for *P. falciparum* was observed at 440 and 543 nm*. P. falciparum* also showed a higher absorbance than media at both 1450 and 1960 nm. To further investigate the spectra, we plotted the 2^nd^ derivative of the average spectra of *P. falciparum*. Peaks related to pure *P. falciparum* were seen at 514, 528, 547, 561, 582 and 595 within the visible region 1388, 1432, 1681, 1700, 1721, 1882, 1905, 2245, 2278 and 2300 nm within the NIR region (Fig 4B). Some of the wavelengths observed to be unique to *P. falciparum* were found within the visible light region at 514, 528, 547, 561, 582 and 595 nm. The highest peaks for *P. falciparum* in this region were at 582 and 595 nm (Fig 4B). Comparatively, no peaks were observed for media used to grow *P. falciparum* in the visible region.

### Summary of *P. falciparum* signature peaks

Six peaks observed for *P. falciparum* belong to the visible light region, two within the NIR 2^nd^ overtone region, five in the 1^st^ overtone region and 3 in the combination band region. The majority of the peaks identified belong to the visible light region and the 1^st^ overtone region (Table 5).

**Table 5:** Summary of peaks identified for *P. falciparum* and their respective functional groups relative to literature [42–48].

Wavelength (nm)	Wavelength region	Functional group/ vibration
514	Visible light	Cyan
528		Green
	547	
	561	
582		Yellow
595		Orange
1388	2^nd^ overtone region	CH_3_
1432		R-OH
1681	1^st^ overtone region	Ar-CH
1700		CH_2_/CH_3_
1721		CH/CH_2_
1882		H_2_O
1905		P-O-H
2245	Combination band region	CH_3_
2278		CH_2_/CH_3_
2300		CH/CH_2_/CH_3_

## Discussion

The aim of this study was to identify visible and NIR peaks that are unique to DENV, RRV, BFV SINV and *P. falciparum* which could serve as potential diagnostic biomarkers for these pathogens. A total of 9 peaks of interest were identified for these arboviruses (Table 2). Distinct signature peaks for BFV were seen at 562, 588, 946, 958 and 1154 nm. Peaks at 562 and 588 nm are within the visible region and could be useful for identification of BFV. Peaks at 946, 958 and 1154 nm represent lipid molecular structures. The lipids could be due to the presences of a bi-lipid membrane anchored on the surface of BFV by proteins E1 and E2 [49].

Besides BFV, the rest of the peaks observed were all shared among the three arboviruses and a peak at 410 nm was observed in all arboviruses. This peak at 410 nm is commonly used for assays that require fluorescent excitation such as ELISA and microscopy [50–53]. DENV and RRV have been identified using this peak previously using a spectrofluorometer [54] and ELISA [55], respectively. A peak at 430 nm was observed for BFV strains and RRV but not DENV and SINV indicating the likelihood that visible light could possibly be used to distinguish between these arboviruses. Peaks at 1130 and 1780 nm were present in BFV strains and RRV. The peak at 1130 nm represents CH_3_ functional group whereas the peak at 1780 nm represents C=O functional group. Both wavelengths indicate the presence of lipid biomolecules [42–47]. For BFV, this peak could be due to the presence of the bi-lipid membrane [49]. RRV uses lipid droplet biogenesis for viral replication [56] and lipid rafts for infection [57] thus these lipids could be the residue from these processes. The peak at 1130 nm was also observed for DENV. Lipids present in DENV could be a by-product from cellular passaging of DENV in C6/36 *Ae. albopictus* cells which use lipid metabolism for efficient replication [58–60]. In addition, we identified this peak in a previous study that detected DENV1 in human blood plasma [61].

To further evaluate if the NIRS spectra could be used to differentiate between arboviruses and media, machine learning algorithms were run on the visible-NIR spectral signatures collected. Using the ANN model, the sensitivity and specificity of ≥90% and 100%, respectively were achieved for the independent test set when samples were grouped as infected or not infected. Sensitivity for predicting arboviruses into their actual group was 100%, 80% and 70% for RRV, DENV and BFV, respectively. Seven out of 40 DENV samples were predicted as BFV. Similarly, 3 out of 20 BFV samples were predicted as DENV (Table 3). This indicates a slight confusion by the training model in differentiating DENV and BFV which could be due to the shared absorption peaks at 410 and 1130 nm.

A total of 16 peaks were identified for *P. falciparum.* Six of those peaks belong to the visible region. This is not surprising as the parasite can be detected via light microscopy. Three of the 6 peaks within the visible region (547, 561 and 582 nm), were also identified at 540, 560 and 579 nm in the ring stage of *P. falciparum* in whole blood as reported by Adegoke and colleagues [62]. Of the 10 remaining wavelengths, 3 in the NIR region (1388, 2245 and 2300 nm) represent C-H bond vibrations [42–47]. C-H bonds are basic chemical building blocks of life and could be responsible for numerous structures within the *P. falciparum* parasite. Four wavelengths of interest (1681, 1700, 1721, 1905 nm) represent lipids [42–48]. Lipids have been found in *P. falciparum* and have been shown to play several roles such as toxicity [63], gametocytogenesis [64], and parasite development [65–68]. In addition, peaks at 1388 and 1432 nm are within the same range as those recently identified (1377 and 1431 nm) in malaria infected patients non-invasively using a handheld spectrometer [25]. The remaining 3 wavelengths; 1432, 1882, 2278 nm represent aromatic amine, water and polysaccharides, respectively [42–48].

The peaks for arboviruses and *P. falciparium* identified in this study will be useful biomarkers for the surveillance/diagnosis of these pathogens in the real world either in humans hosts, animal hosts or mosquito vectors. However, a further assessment in the field using naturally infected blood samples is required to validate the biomarkers identified under this study. With this additional tool in hand, NIRS has the potential to rapidly identify infections to stop an outbreak by facilitating timely isolation and treatment of patients and rapid identification of infected mosquitoes.

## Conclusion

We have identified several novel visible and NIR biomarkers for BFV, DENV, RRV, SINV and *P. falciparum*. To our knowledge, this is the first investigation to report NIR biomarkers for arboviruses. The findings of this study provide insights into the potential future application of these peaks as diagnostic biomarkers for these pathogens. Future work should evaluate the capability of NIRS spectrometers coupled with machine learning to detect these pathogens, using these biomarkers, in human, animal and mosquito species. This would facilitate rapid, non-invasive, and cost-effective diagnosis and surveillance of these pathogens particularly in large-scale setting that require programmatic surveillance to stop outbreaks.

## Supporting information

Table S1 A summary of the validation set from ANN testing of different BFV strains, DENV serotypes and SINV. Overall, a misclassification rate of 0.4778 (n=90) was obtained indicating a low overall accuracy. Numbers in the table represent the number of technical replicates allocated to the prediction count for each arbovirus and media.
(DOCX)

Table S2 A summary of statistical models tested with training data. Statistical models are ranked in order of most accurate to the least. Accuracy is determined by the model having values: lowest for misclassification rate, highest for entropy RSquare, highest for area under the curve, lowest for root average square error, and highest for generalized RSquare.
(DOCX)

Fig S1 The average visible-NIR raw spectra of DENV media (A), BFV/media (B), RRV/media (C) and SINV/media (D). Prominent NIRS peaks for DENV can be observed in the 1450 nm and 1950 nm regions and water peaks were identified at 1450 and 1950 nm. Generally, the absorbance value of DENV was lower than that of media (A). All spectra of BFV QML and BFV WEN1631 were averaged to identify prominent absorbance peaks for BFV. Generally, BFV was observed to have higher absorbance values than media (B). The absorbance value of RRV within the visible and NIR regions was generally higher than media except for the water peak around 1940 nm (C). SINV 18953 generally absorbed less light than media from the visible through to the NIR region between 350–2500 nm. Absorbance values for water molecules at 1450 and 1950 nm of SINV 18953 was lower than media (D).
(DOCX)

Fig S2 The 2^nd^ derivative of the averaged visible and NIR spectra of arboviruses and media. The 2^nd^ derivative of the average visible and NIR spectra for DENV/media (A), BFV/Media (B), RRV/Media (C) and SINV (D) from 350–2500 nm.
(DOCX)

S1 Supporting Information Data All data for this study is included in the file titled “Supporting information data”. (XLSX)
