# Supplementary material for: Identification of visible and near-infrared signature peaks for arboviruses and Plasmodium falciparum
Source: PLoS One. 2025 Apr 17;20(4):e0321362. doi: 10.1371/journal.pone.0321362 (PMC12005544; doi:10.1371/journal.pone.0321362)
Supplement: Table S1 — Overall, a misclassification rate of 0.4778 (n=90) was obtained indicating a low overall accuracy. Numbers in the table represent the number of technical replicates allocated to the prediction count for each arbovirus and media. (DOCX) [file pone.0321362.s001.docx]

| Validation set | Prediction count | | | | | | | | | |
| --- | --- | --- | --- | --- | --- | --- | --- | --- | --- | --- |
| Arbovirus | BFV QML | BFV WEN | RRV QML1 | SINV 18953 | DENV1 EM-093 | DENV2 NGC | DENV3 44002 | DENV4 AFRIM | Media |  |
| BFV QML (n=10) | 7 | 0 | 0 | 3 | 0 | 0 | 0 | 0 | 0 |  |
| BFV WEN (n=10) | 2 | 6 | 0 | 2 | 0 | 0 | 0 | 0 | 0 |  |
| RRV QML1  (n=10) | 0 | 0 | 5 | 0 | 1 | 0 | 3 | 0 | 1 |  |
| SINV 18953  (n=10) | 0 | 0 | 0 | 2 | 0 | 0 | 0 | 8 | 0 |  |
| DENV1 EM-093  (n=10) | 4 | 0 | 0 | 0 | 5 | 1 | 0 | 0 | 0 |  |
| DENV2 NGC  (n=10) | 0 | 0 | 0 | 0 | 9 | 1 | 0 | 0 | 0 |  |
| DENV3 44002  (n=10) | 0 | 0 | 0 | 1 | 0 | 0 | 9 | 0 | 0 |  |
| DENV4 AFRIM  (n=10) | 2 | 0 | 0 | 1 | 0 | 0 | 0 | 7 | 0 |  |
| Media  (n=10) | 0 | 0 | 2 | 3 | 0 | 0 | 0 | 0 | 5 |  |

Table S1. A summary of the validation set from ANN testing of different BFV strains, DENV serotypes and SINV. Overall, a misclassification rate of 0.4778 (n=90) was obtained indicating a low overall accuracy. Numbers in the table represent the number of technical replicates allocated to the prediction count for each arbovirus and media.
