# Supplementary material for: Identification of visible and near-infrared signature peaks for arboviruses and Plasmodium falciparum
Source: PLoS One. 2025 Apr 17;20(4):e0321362. doi: 10.1371/journal.pone.0321362 (PMC12005544; doi:10.1371/journal.pone.0321362)
Supplement: Table S2 — Statistical models are ranked in order of most accurate to the least. Accuracy is determined by the model having values: lowest for misclassification rate, highest for entropy RSquare, highest for area under the curve, lowest for root average square error, and highest for generalized RSquare. (DOCX) [file pone.0321362.s002.docx]

Table S2. A summary of statistical models tested with training data. Statistical models are ranked in order of most accurate to the least. Accuracy is determined by the model having values: lowest for misclassification rate, highest for entropy RSquare, highest for area under curve, lowest for root average square error, and highest for generalised RSquare.

| Method | Misclassification rate | Entropy RSquare | Area under curve | Root average square error | Generalised RSquare |
| --- | --- | --- | --- | --- | --- |
| Neural Boosted | 0.0035 | 0.9146 | 1 | 0.13873 | 0.9765 |
| Bootstrap Forest | 0.0767 | 0.7587 | 0.9972 | 0.29048 | 0.9195 |
| Support vector machines | 0.1953 | 0.3951 | 0.9221 | 0.41704 | 0.7868 |
| Naïve Bayes | 0.3628 | -1.881 | 0.6764 | 0.60232 | -90.57 |
