# Supplementary material for: Identification of visible and near-infrared signature peaks for arboviruses and Plasmodium falciparum
Source: PLoS One. 2025 Apr 17;20(4):e0321362. doi: 10.1371/journal.pone.0321362 (PMC12005544; doi:10.1371/journal.pone.0321362)
Supplement: Fig S1 — Prominent NIRS peaks for DENV can be observed in the 1450 nm and 1950 nm regions and water peaks were identified at 1450 and 1950 nm. Generally, the absorbance value of DENV was lower than that of media (A). All spectra of BFV QML and BFV WEN1631 were averaged to identify prominent absorbance peaks for BFV. Generally, BFV was observed to have higher absorbance values than media (B). The absorbance value of RRV within the visible and NIR regions was generally higher than media except for the water peak around 1940 nm (C). SINV 18953 generally absorbed less light than media from the visible through to the NIR region between 350–2500 nm. Absorbance values for water molecules at 1450 and 1950 nm of SINV 18953 was lower than media (D). (DOCX) [file pone.0321362.s003.docx]

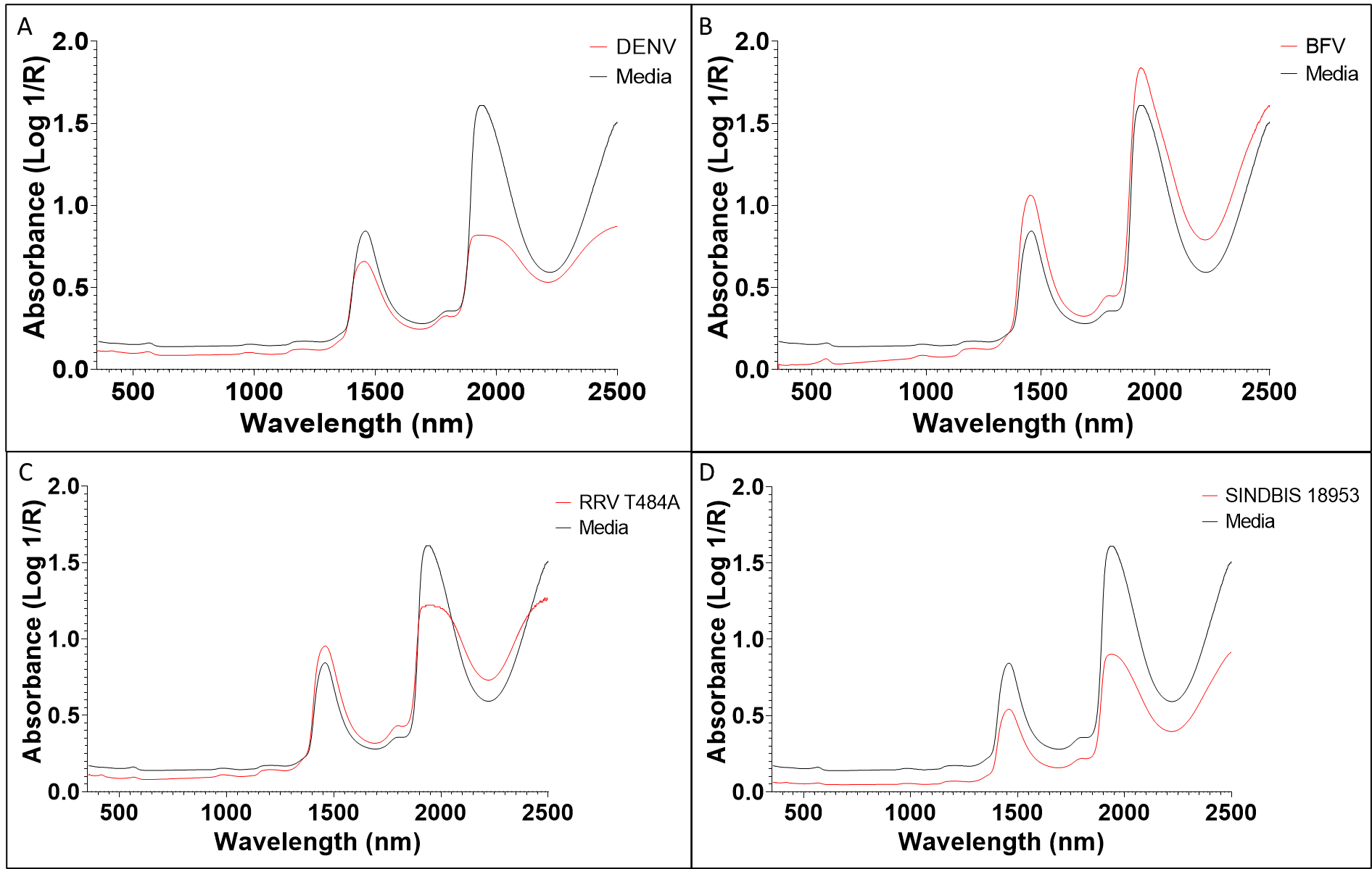


Figure S3. The average visible-NIR raw spectra of DENV media (A), BFV/media (B), RRV/media (C) and SINV/media (D). For DENV, prominent NIRS peaks can be observed in the 1450 nm and 1950 nm regions and water peaks were identified at 1450 and 1950 nm. Generally, the absorbance value of DENV was lower than that of media (A). All spectra of BFV QML and BFV WEN1631 were averaged to identify prominent absorbance peaks for BFV. Generally, BFV was observed to have higher absorbance values than media (B). The absorbance value of RRV within the visible and NIR regions was generally higher than media except for the water peak around 1940 nm (C). SINV 18953 generally absorbed less light than media from the visible through to the NIR region between 350- 2500 nm. Absorbance values for water molecules at 1450 and 1950 nm of SINV 18953 was lower than media (D).
