# Supplementary material for: Identification of visible and near-infrared signature peaks for arboviruses and Plasmodium falciparum
Source: PLoS One. 2025 Apr 17;20(4):e0321362. doi: 10.1371/journal.pone.0321362 (PMC12005544; doi:10.1371/journal.pone.0321362)
Supplement: Fig S2 — The 2nd derivative of the average visible and NIR spectra for DENV/media (A), BFV/Media (B), RRV/Media (C) and SINV (D) from 350–2500 nm. (DOCX) [file pone.0321362.s004.docx]

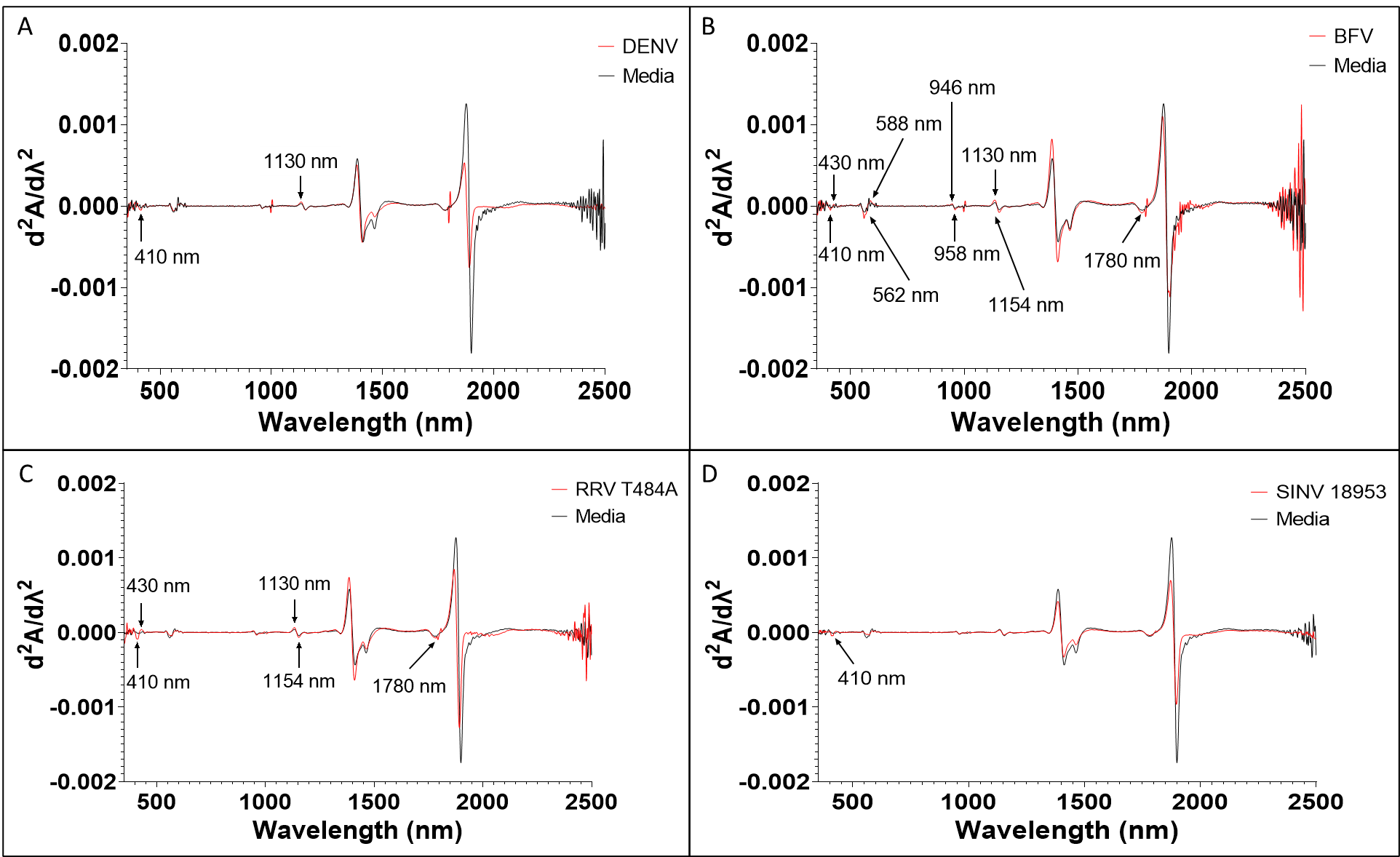


Figure S4. The 2^nd^ derivative of the averaged visible and NIR spectra of arboviruses and media. The 2^nd^ derivative of the average visible and NIR spectra for DENV/media (A), BFV/Media (B), RRV/Media (C) and SINV (D) from 350-2500 nm.
